# Divergent Mechanisms Controlling Hypoxic Sensitivity and Lifespan by the DAF-2/Insulin/IGF-Receptor Pathway

**DOI:** 10.1371/journal.pone.0007937

**Published:** 2009-11-20

**Authors:** Meghann E. Mabon, Barbara A. Scott, C. Michael Crowder

**Affiliations:** 1 Department of Anesthesiology, Washington University School of Medicine, St. Louis, Missouri, United States of America; 2 Department of Developmental Biology, Washington University School of Medicine, St. Louis, Missouri, United States of America; 3 The Division of Biology & Biomedical Sciences, Program in Developmental Biology, Washington University School of Medicine, St. Louis, Missouri, United States of America; New Mexico State University, United States of America

## Abstract

Organisms and their cells vary greatly in their tolerance of low oxygen environments (hypoxia). A delineation of the determinants of hypoxia tolerance is incomplete, despite intense interest for its implications in diseases such as stroke and myocardial infarction. The insulin/IGF-1 receptor (IGFR) signaling pathway controls survival of *Caenorhabditis elegans* from a variety of stressors including aging, hyperthermia, and hypoxia. *daf-2* encodes a *C. elegans* IGFR homolog whose primary signaling pathway modulates the activity of the FOXO transcription factor DAF-16. DAF-16 regulates the transcription of a large number of genes, some of which have been shown to control aging. To identify genes that selectively regulate hypoxic sensitivity, we compared the whole-organismal transcriptomes of three *daf-2* reduction-of-function alleles, all of which are hypoxia resistant, thermotolerant, and long lived, but differ in their rank of severities for these phenotypes. The transcript levels of 172 genes were increased in the most hypoxia resistant *daf-2* allele, *e1370*, relative to the other alleles whereas transcripts from only 10 genes were decreased in abundance. RNAi knockdown of 6 of the 10 genes produced a significant increase in organismal survival after hypoxic exposure as would be expected if down regulation of these genes by the *e1370* mutation was responsible for hypoxia resistance. However, RNAi knockdown of these genes did not prolong lifespan. These genes definitively separate the mechanisms of hypoxic sensitivity and lifespan and identify biological strategies to survive hypoxic injury.

## Introduction

Low ambient oxygen concentrations can induce cell death. However, cells vary greatly in the level and duration of hypoxia that is required to produce their death. Even within the same organism hypoxic sensitivity can range widely. In humans, neurons and cardiac myocytes are exquisitely hypoxia sensitive, which can lead to stroke and myocardial infarction. Thus, identification of the determinants of hypoxic sensitivity has both biological and medical significance.

We have previously shown that reduction-of-function mutations in the *daf-2* gene confer powerful protection from hypoxic injury in the nematode *Caenorhabditis elegans* [1]. *daf-2* encodes an insulin/IGF-receptor homolog that signals through a conserved PI-3 kinase signaling cascade to regulate negatively the activity of a FOXO transcription factor, DAF-16 [2], [3]. *daf-2* reduction-of-function mutants are not only hypoxia resistant but are also thermotolerant, long lived, and have a propensity to form dauer larvae, which are capable of long periods of hibernation in response to environmental stress [4]. Because of the very interesting biological processes controlled by the *daf-2 – daf-16* pathway, considerable effort has been made to identify the downstream targets of the pathway [5], [6], [7], [8], [9], [10], [11], [12]. The various methods used in these studies along with the different phenotypes screened for produced only modestly overlapping gene sets. Thus, DAF-16 appears directly or indirectly to control the expression of a large number of diverse genes that often influence only a specific *daf-2* phenotype.

Multiple *daf-2* alleles have been isolated, primarily in screens for mutants that constitutively form dauers. Gems et al. measured phenotypes for sixteen *daf-2* alleles and found lack of concordance for the severity of the different phenotypes in several cases [13]. For example, *daf-2(e1370)* is moderately long lived and dauer constitutive but was the most thermotolerant of all alleles tested. On the other hand, *daf-2(e1368)* was considerably less thermotolerant and dauer constitutive than *e1370* but was found to have a median lifespan about 50% longer than *e1370*. We measured the hypoxic sensitivity of thirteen *daf-2* alleles and found a wide range of sensitivities [1]. For example, *e1370* was the most hypoxia resistant *daf-2* allele tested while *e1368* was very weakly resistant. The mechanism underlying this divergence of phenotypic severities among alleles is obscure.

In the current study utilizing cDNA microarrays and double stranded RNA-mediated interference (RNAi) of the expression of candidate genes, we take advantage of the *daf-2* allelic variations to identify genes regulated by *daf-2* that selectively alter hypoxic sensitivity without affecting lifespan. The identity of these genes implicates novel pathways for the selective regulation of hypoxic sensitivity.

## Results

### *daf-2* Alleles Reveal Divergent Outputs for Hypoxic Survival and Lifespan

**Figure 1:**
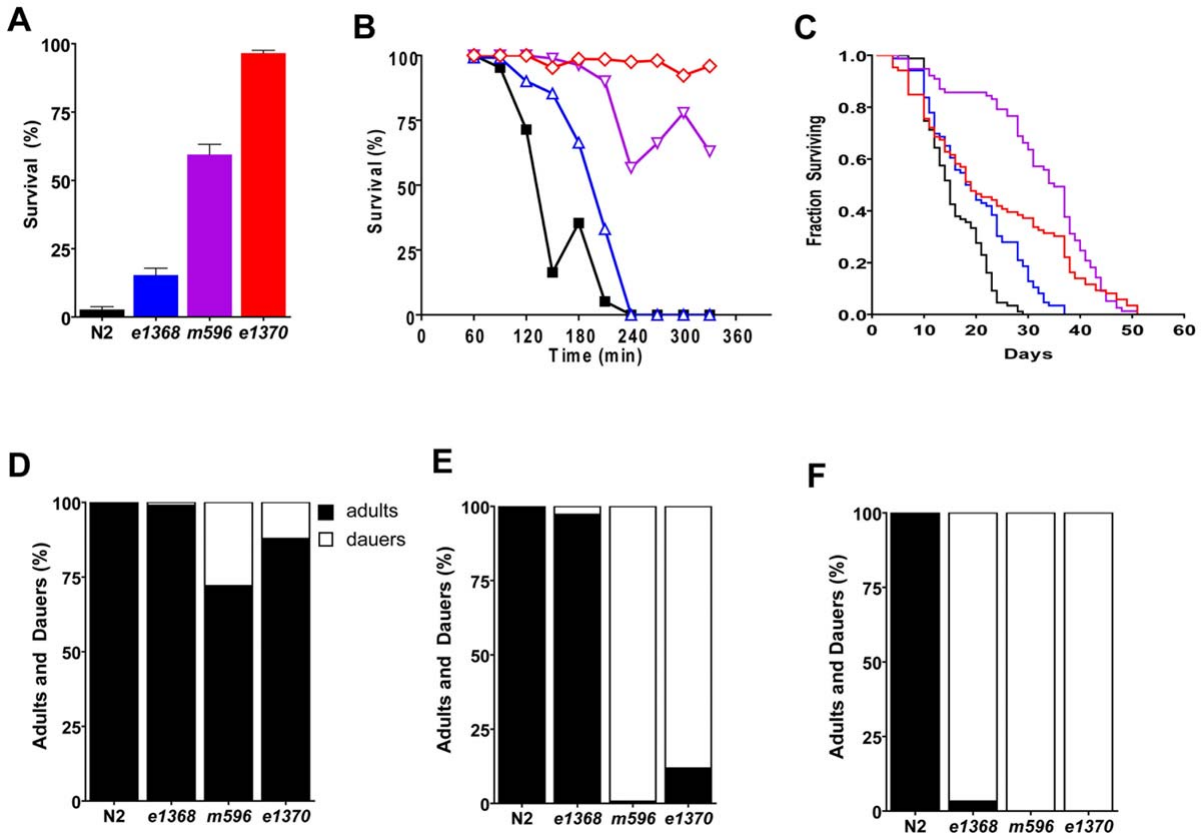
*daf-2* allelic series for hypoxia resistance does not correlate with all *daf-2* phenotypes. (A) Survival following recovery from 20 hour hypoxia insult (plotted data are mean±SD of at least four independent experiments). (B) Wild-type (N2) and *daf-2* mutants were assayed for thermotolerance (mean of three trials is plotted); *e1370* (red line), *m596* (purple line), *e1368* (blue line) and N2 (black line). (C) Lifespans of N2 and three *daf-2* alleles. Each survival curve was generated from a minimum of 77 animals. Animals that bagged or crawled from the plate were censored. (D, E, F) Dauer arrest was measured at 20° (C), 23° (D), and 25° in N2 and *daf-2* mutants. Black bars represent adults and white bars represent dauers. All strains were statistically different from one another for hypoxic survival, thermotolerance, and dauer formation (except N2 and *e1368* in D,E and *m596* and *e1370* in F), p<0.01 (two-tailed t-test for A, linear regression for B, Mantel-Cox Log-rank test for C, Fisher's exact χ^2^ for D, E, and F.

We chose three *daf-2* alleles for our study *e1370, m596*, and *e1368*. We previously reported that after recovery from 20 hours of hypoxic incubation, whole organism survival for these alleles was 96%, 53%, and 23%, respectively, compared to 4% for the wild type strain N2 [1]. Gems et al. scored the dauer constitutive, thermotolerance, and long lifespan phenotypes of these alleles [13]. However, the severities of *daf-2* phenotypes have varied considerably among labs, particularly for lifespan. Thus, we scored the lifespan, thermotolerance, dauer constitutive, and hypoxia resistant phenotypes of all three *daf-2* alleles contemporaneously to confirm their phenotypic divergence. We again found that *e1370* was strongly hypoxia resistant, *m596* moderately resistant, and *e1368* weakly so (Figure 1A). The order of severities for the alleles was quite similar for thermotolerance (Figure 1B). However, for lifespan *m596* was the strongest allele having a mean lifespan of 31.3 days compared to 22.5 days for *e1370* and 18.8 for *e1368* (Figure 1C and Table 1). For the dauer constitutive phenotype, *m596* was the most severe allele; *e1370* was a close second while *e1368* was a much weaker allele (Figure 1D,E,F). The results are generally consistent with those previously reported by Gems et al. [13]. These authors reported that *e1370* was most thermotolerant, followed closely by *m596*, and more distantly by *e1368*. At a culture temperature of 22.5°C, *m596* had the longest lifespan followed by *e1368* and then *e1370*. For the dauer constitutive phenotype, the order of severity was as we found: *m596*>*e1370*>*e1368*.

**Table 1:** Lifespan analysis of *daf-2* strains.

Strain	Mean (d)	Maximum (d)	n
N2	14.9	29	87
*Daf-2(e1368)*	18.8*	37*	86
*Daf-2(m596)*	31.3*	51*	77
*Daf-2(e1370)*	22.5* †	51*	86

Day 1 begins at first day of adulthood.

* p<0.01 vs N2;

† - p<0.01 vs e1368 and m596.

### Transcriptional Profiling of *daf-2* Alleles

**Figure 2:**
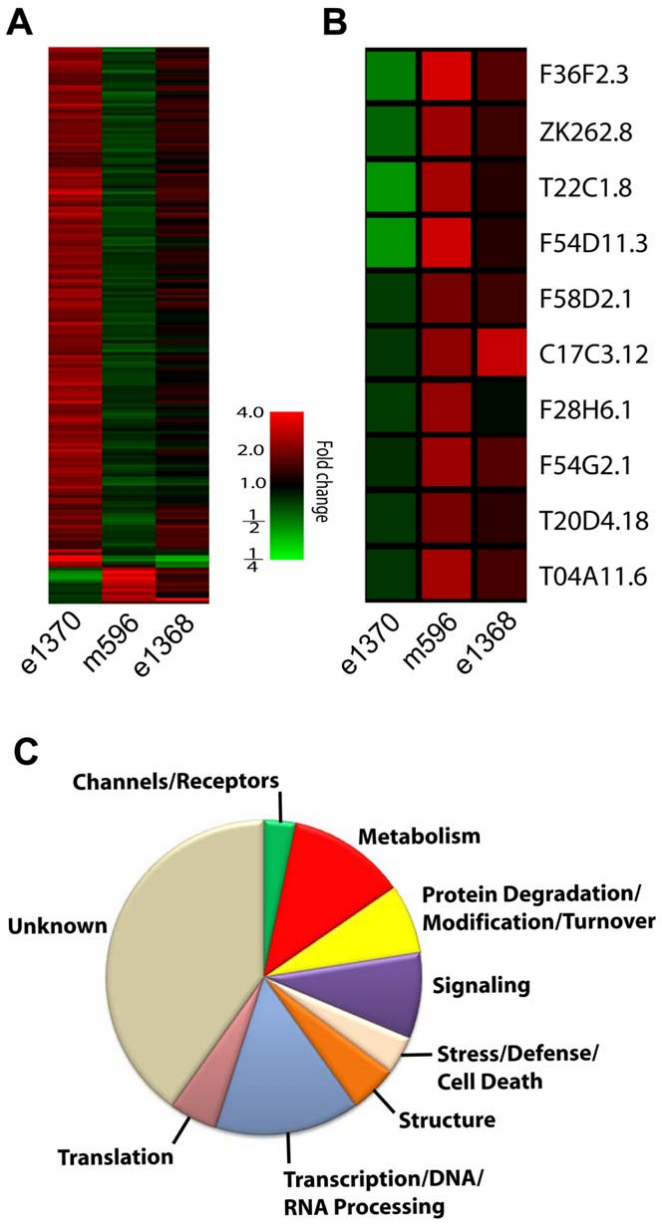
Expression of 182 genes are differentially regulated across *daf-2* alleles. (A) Heat map of expression of 182 genes, either up- or down-regulated between *e1370* and *m596* and/or *e1368*, p<0.01, One-Way ANOVA. (B) Genes whose expression is downregulated in *e1370* (*die*) relative to *m596* and/or *e1368*, p<0.01. (C) Relative number of *daf-2* regulated genes that fall into the various functional categories.

The transcriptional output of the *daf-2 - daf-16* pathway and the distinct *daf-2* allelic series for hypoxia versus lifespan and dauer formation offer a means to identify genes in the *daf-2 - daf-16* pathway that specifically regulate hypoxia. We hybridized whole genome oligonucleotide microarrays with cDNA derived from *e1370, m596*, or *e1368* L4 stage larval animals grown under normal conditions. Comparisons of the transcriptome across alleles revealed 182 genes that were statistically different (p<0.01) between *e1370* and *m596* and/or *e1368* (Figure 2A); Raw data have been deposited in the NCBI Gene Expression Omnibus database (http://www.ncbi.nlm.nih.gov/geo/), accession number GSE18601. 172 genes were up-regulated in *e1370* compared to *m596* and/or *e1368* (Table S1), and 10 were relatively down-regulated in *e1370* (Table 2, Figure 2B). The vast majority of the *e1370*-upregulated genes had a greater increase in expression relative to *m596* than relative to *e1368*. These results suggest that the majority of these genes may regulate lifespan, a trait where *e1370* diverges more from *m596* than *e1368*. The expression of only six of the 182 genes followed the hypoxic phenotypic allelic series, that is either up- or down-regulated in the order of *e1370, m596, e1368* (Table 3). Of these, only one, C17C3.12, encodes a strong mammalian homolog, a mitochrondrial acyl-CoA dehydrogenase, which functions catabolically in mammals in fatty acid oxidation. In general, genes involved in regulation of metabolic processes were highly represented in the 182 gene list (Figure 2C). Transcriptional/DNA/RNA processing genes were also abundant.

**Table 2:** Genes transcriptionally down-regulated in *e1370* vs *m596* or *e1368* – *die* genes.

Sequence Name	Gene Name	Protein/Homology	Biological Process	Expression	p-value	*e1370*vs *m596*	*e1370*vs *e1368*	*e1368*vs *m596*
F36F2.3		Predicted E3 ubiquitin ligase	Protein Degradation/Modification/Turnover	ND	0.0019	−6.0	−2.6	−2.3
ZK262.8		synthetic lethal with Argonaute mutant	Unknown	ND	0.0021	−3.6	−1.9	−1.9
T22C1.8		Protein tyrosine phosphatase	Signaling	ND	0.0027	−5.2	−2.5	−2.1
F54D11.3		*C. elegans*-specific	Unknown	ND	0.0048	−7.1	−2.6	−2.7
F58D2.1		ZYG-11-like serine/threonine protein kinases	Unknown	ND	0.0059	−2.2	−1.5	−1.5
C17C3.12	*acdh-2*	Mitochondrial short-chain acyl-CoA dehydrogenase	Metabolism	ND	0.0060	−2.4	−3.6	−1.5
F28H6.1	*akt-2*	Homolog of the serine/threonine kinase Akt/PKB	Signaling	Intestine, Neuronal, Hypodermal, Pharyngeal	0.0061	−2.7	−1.3	−2.1
F54G2.1		Isoform 1 of protein UNC-13 homolog D	Unknown	Intestine, Neuronal	0.0072	−2.5	−1.6	−1.6
T20D4.18	*srab-21*	Sra family integral membrane protein	Unknown	Hypodermal, Neuronal	0.0077	−2.1	−1.4	−1.5
T04A11.6	*him-6*	ATP-dependent DNA helicase	Transcription/DNA/RNA Processing	Primarily Germline	0.0086	−2.9	−1.5	−1.9

Annotations from WormBase (www.wormbase.org); p-value one-way ANOVA for *e1370* vs *m596*.

**Table 3:** Genes regulated according to the allelic series.

Sequence Name	Common Name	*e1370* *	*m596* *	*e1368* *
C14A4.8		2.19	0.90	0.85
Y37H2A.6	*fbxa-211*	1.20	0.92	0.90
F45D11.4		3.64	0.62	0.34
F45D11.14		3.70	0.54	0.28
C17C3.12	*acdh-2*	0.79	1.88	2.86
T26H5.1		0.83	1.17	1.20

* Mean of normalized expression, p<0.01.

A number of previous studies have identified genes whose expression is regulated by *daf-2* [7], [8], [9], [10], [11], [12], [14], [15]. These studies have implicated a large number of genes as being regulated by the *daf-2 - daf-16* pathway. However, the overlap among the datasets is relatively modest presumably due to the distinct methodologies employed by each study. Perhaps because of the experimental design of the microarrays that sought gene expression differences among *daf-2* alleles, the overlap between the 182 genes identified here and those from the other studies was also modest with only seven genes in common (Table 4). Four of the seven genes had expression changes that were qualitatively similar to those found previously. For example, the Y54G11A.13/*ctl-3* gene encodes one of three *C. elegans* catalases that are predicted to reduce peroxide free radicals. The *ctl-3* transcript is increased in *e1370* compared to both *e1368* and *m596*. Likewise using quantitative mass spectrometry methods, Dong et al. found that CTL-3 protein was increased in *e1370* compared to wild type worms [14]. Similarly, both our data and that from Murphy et al. [9] show increased expression in *e1370* mutants of *unc-38*, which encodes a subunit of a nicotinic acetylcholine receptor.

**Table 4:** Genes that overlap with other datasets.

Sequence Name	Overlapping Study	Expression in *e1370* relative to *m596, e1368*	Expression in *e1370* relative to wild type*
C09G4.5	Murphy	Increased	Decreased
C47E8.5	Dong	Increased	Decreased
F15E11.1	Dong	Increased	Increased
F17C11.9	Dong	Increased	Decreased
F21F3.5	Murphy	Increased	Increased
F45D11.14	Dong	Increased	Increased
Y54G11A.13	Dong	Increased	Increased

Murphy et al. *Nature 424*∶277-83, 2003. Dong et al. *Science 317*∶660-3, 2007.

* - data from overlapping study.

Ten genes were **d**own-regulated **i**n ***e*** *1370* (*die*) compared to the other alleles (Table 2, Figure 2B). These genes include *akt-2*, a homolog of the serine/threonine protein kinase Akt/PKB. *akt-2* along with its paralog, *akt-1*, functions downstream of *daf-2* in the *daf-2 - daf-16* pathway. Knockdown of *akt-1* and *akt-2* produce a partially redundant dauer constitutive phenotype [16], and an *akt-1* gain-of-function mutant partially suppresses the hypoxia resistance of *daf-2(1370)* [1]. The role of AKT-1/2 downstream of DAF-2 is thought to be through phosphorylation of DAF-16, thereby inhibiting its nuclear localization and transcription factor activity [17], [18]. These new data suggest that a positive feedback loop may exist that enhances the reduction-of-function phenotype of *e1370* compared to *m596* and *e1368* by reducing the levels of *akt-2* mRNA. Besides *akt-2*, two other *die* genes are predicted to have kinase or phosphatase activity, but their substrates are unknown. Other particularly interesting *die* genes include *acdh-2*, which as noted above has expression differences in the alleles that follow the hypoxic sensitivity allelic series, F54G2.1 - an isoform of the presynaptic vesicle fusion protein UNC-13, F36F2.3 - an E3 ubiquitin ligase, and T04A11.6 - an ATP-dependent DNA helicase. We previously found in a whole-genome RNAi screen for genes that promote hypoxic death that knockdown of several genes, predicted to control chromatin structure, also produce significant hypoxia resistance [19]. The current data show that the *daf-2* pathway acts in part through regulation of DNA/chromatin modifying enzymes to control hypoxic sensitivity. However, it is unclear how this protects the organism from hypoxic injury.

### Increased Hypoxic Survival by *die* Gene Knockdown

Genes whose expression was increased in *e1370* relative to the other two alleles are candidates to encode hypoxia protective factors. We chose 10 genes with the highest normalized relative fold change in *e1370* compared to *m596* to test for hypoxia protection (Table S1). We used feeding RNAi to reduce their expression in an *e1370* background with the prediction that this would suppress at least partially its hypoxia resistant phenotype. However, we did not observe a significant change in hypoxia resistance with treatment by any of these RNAis (data not shown). This negative result could be explained in a number of ways. Obviously, inadequate knockdown of gene expression is one way. Given that 172 genes were found to be up-regulated, another likely possibility is that knockdown of no single gene is sufficient to produce a measurable phenotype. Alternatively, the up-regulated genes that were tested could mediate responses either maladaptive or unrelated to survival from hypoxia; thus, knockdown would not suppress *e1370*'s hypoxia resistance.

**Figure 3:**
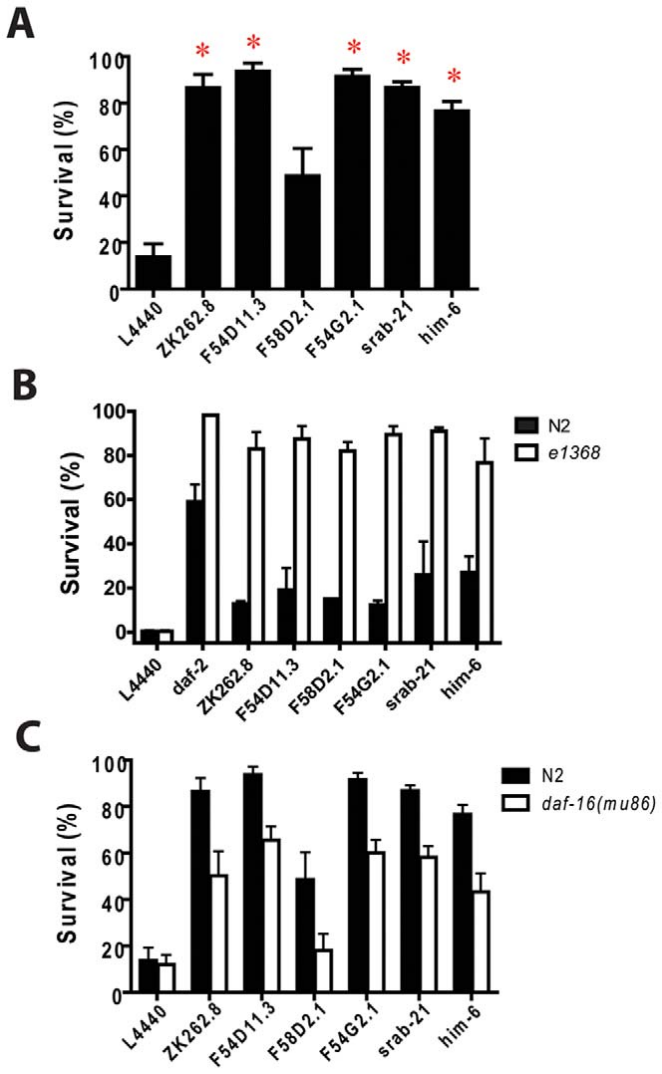
Survival from hypoxia by RNAi knockdown of *die* genes. Genetic background of strains is indicated by closed (wild type - N2) or open bars (mutant – either *daf-2(e1368)* or *daf-16(mu86)*). Note that survival data are after a 22 hour hypoxic incubation for the upper and lower panels and are after a 24 hour hypoxic incubation for the middle panel. (A) Survival after recovery from a 22 hour hypoxic incubation for N2 wild type animals treated with bacteria transfected with L4440 empty vector or with RNAi plasmids against *die* genes. * p<0.01 compared to L4440 (two-tailed t-test, 6 trials/RNAi with >30 worms/trial). (B) Effect of RNAi treatments on survival after a 24 hour hypoxic incubation in wild type and *daf-2(e1368)* backgrounds. All RNAi's resistance in an e1368 background compared to L4440 – p<0.01 (two-tailed t-test, 3 trials/RNAi with >30 worms/trial) (C) Effect of RNAi treatments on survival after a 22 hour hypoxic incubation in wild type and *daf-16(mu86* null) backgrounds. Hypoxia resistance significantly suppressed in *mu86* background for all RNAi's – p<0.01 (paired t-test, 6 trials/RNAi with >30 worms/trial).

The *die* genes are candidates to promote *daf-2*-regulated hypoxic sensitivity; thus, *die* gene reduction-of-function might produce hypoxia resistance. To test this hypothesis, we used RNAi to knockdown six of the ten *die* genes; of the four not tested, two, C17C3.12 and F28H6.1, were not in our library, one other, T22C1.8, did not grow when streaked, and the fourth, F36F2.3, produced sick animals in the absence of hypoxia. Five of six RNAi treatments conferred significant hypoxia resistance to otherwise wild type animals (Figure 3A). We then tested the effect of knockdown of the six *die* genes in an *e1368* background. Knockdown of all six genes produced strong hypoxia resistance in an *e1368* background compared to the empty vector control (Figure 3B). These results show that reduction of function of any one of these genes is capable of converting the weakly hypoxia resistant *e1368* animals to strongly resistant ones. Also, the sensitized background of *e1368* revealed that F58D2.1 does in fact encode a hypoxic sensitivity promoting activity that was not apparent in a wild type background.

Given that expression of *die* gene transcripts is regulated by *daf-2*, we hypothesized that the hypoxia resistance phenotype of *die* RNAi may depend on DAF-16, the primary target of DAF-2. Indeed, the hypoxia resistance phenotypes of all the *die* genes were suppressed by a null mutation in *daf-16* (Figure 3C). However, the suppression of the hypoxia resistance was incomplete for five of the six RNAi knockdowns. Thus, these genes do not regulate hypoxic sensitivity entirely via a DAF-16 dependent pathway.

### *die* Genes Are Not Equally Required for Thermotolerance or Lifespan Extension

**Figure 4:**
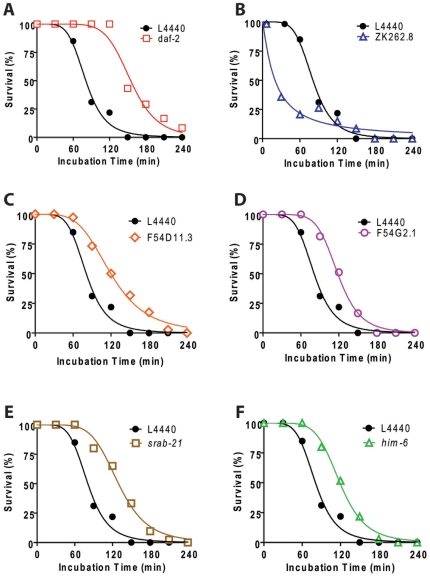
Increased thermotolerance by *die* gene RNAi knockdown. Survival after recovery from incubations at 37° for various times for worms raised on L4440 empty vector or the denoted RNAi. RNAi's in panel A and C-F produce significantly increased thermotolerance whereas the RNAi in panel B produces significantly decreased thermotolerance – p<0.01, F-test by nonlinear regression and simultaneous curve-fitting. A minimum of 353 animals/curve with at least 18 animals/data point were scored.

**Figure 5:**
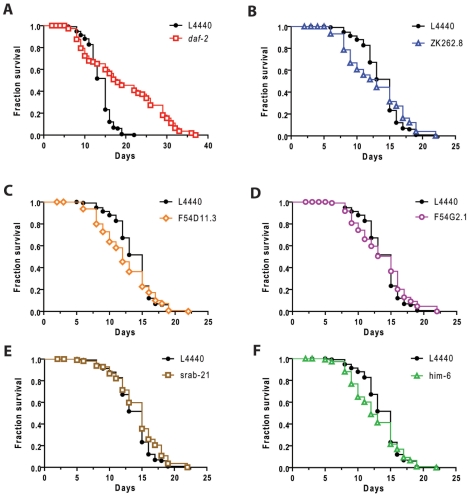
No effects on lifespan by *die* gene RNAi knockdown. Lifespan survival curves for animals grown on L4440 empty vector bacteria versus RNAi bacteria. (A) The effect of *daf-2* RNAi on lifespan is shown as a positive control and is significantly different from L4440 lifespan – p≪0.01 Mantel-Cox Log-rank test. (B-F) None of the other RNAis significantly lengthen lifespan at the p<0.01 level. Lifespan assays were performed with 120 animals/RNAi grown at 20°C.

The experimental design of the microarrays was devised to enrich for genes that selectively regulate hypoxic sensitivity. To test for selective regulation, we measured the thermotolerance and lifespan of the five *die* genes where knockdown produced hypoxia resistance in a wild type background. Knockdown of four of the five *die* genes produced significant thermotolerance (Figure 4). Only ZK262.8 RNAi did not protect from thermal stress (Figure 4B). These results are consistent with the strong correlation between thermotolerance and hypoxia resistance observed among various *daf-2* alleles [1]. However, they also show that the mechanisms of thermal injury and hypoxic injury in *C. elegans* are not identical. Despite a considerable increase in both mean and maximum lifespan by *daf-2* RNAi, none of the *die* gene RNAi's resulted in a significant alteration in lifespan (Figure 5 and Table S2). Again, these results are consistent with the poor correlation between the lifespan and hypoxic sensitivity phenotypes of *daf-2* alleles [1].

One possibility for the strong correlation between thermotolerance and hypoxia resistance is the 26.5°C temperature at which the hypoxic incubations are performed. As in all models of hypoxic injury, the sensitivity of *C. elegans* to hypoxia is temperature dependent [1]; the higher the temperature the more severe the hypoxic injury. However, a number of observations suggest that the hypoxic sensitivity differences among *daf-2* alleles is not simply a manifestation of varied thermotolerance. First, two *daf-2* alleles, *e979* and *e1369* with only moderate hypoxia resistance similar to that of *m596*, have thermotolerances at least as high as *e1370* [13]. Second, when hypoxic incubations are performed at 20°C, *e1370* and *m579*, which is highly hypoxia resistant under our assay conditions [1], are still highly hypoxia resistant, whereas *m596* is not [20]. Finally, we asked whether high temperature incubation in buffer but in a room air environment produced significant wild type animal death that might account for the *daf-2* allelic differences for hypoxic sensitivity at higher temperature. However, we observed no significant death of wild type animals after a 20 hour normoxic incubation at 28°C (Figure S1). Even a 71 hour incubation killed only 5% of animals.

## Discussion

Utilizing *daf-2* alleles with distinct orders of phenotypic severity for hypoxic sensitivity versus its other phenotypes as the basis for DNA microarray experiments, we identified 182 genes that were differentially expressed in animals with varying levels of hypoxia resistance. The expression of most of these genes was increased in the hypoxia resistant *daf-2(e1370)*. These genes are candidates for antagonizing hypoxic death. However, we were unable to detect hypoxic hypersensitivity by RNAi knockdown of a subset of these genes. In general, we have found hypoxic hypersensitivity is difficult to produce in our model; this may reflect genetic redundancy and is reminiscent of the relatively uncommon phenotype of shortened lifespan as compared to prolonged lifespan. However, for the ten genes that were decreased in *daf-2(e1370)*, we were able to test six of them and found that knockdown of each of them produced hypoxia resistance. These genes are of interest not only for the fact that they control hypoxic sensitivity but that they also do it in a relatively specific manner by having no detectable influence on lifespan. In this regard, ZK262.8 is the most intriguing because unlike the other five *die* genes with a hypoxia resistance knockdown phenotype, ZK262.8 knockdown does not confer thermotolerance. ZK262.8 is a poorly characterized gene with no clear homology to genes in other organisms. It was implicated in microRNA processing because of a synthetic lethal phenotype with a mutation in the Argonaut homolog gene, *alg-2* [21]. ALG-2 is expressed in most, if not all, cells in *C. elegans* from embryonic to adult stages and is hypothesized to facilitate loading of pre-miRNAs into the Dicer complex [21]. However, it is unclear what role if any the ZK262.8 gene product has in miRNA processing.

Another hypoxic death promoting *die* gene identified is *srab-21*, which encodes a serpentine receptor that is likely to function in chemosensation [22]. In a whole genome RNAi screen for genes promoting hypoxic death, we identified eleven serpentine receptor genes where knockdown produced hypoxia resistance [19]. These genes represented about 5% of the genes identified in the screen. Given that these genes are thought to encode chemosensory receptors, normal chemosensory function appears to promote hypoxic sensitivity in *C. elegans*. Likewise for aging, mutations in genes required for chemosensation prolong lifespan [23]. The prolonged lifespan of these mutants could be suppressed by mutations in *daf-16*, and a model was proposed whereby activation of chemosensory neurons increases insulin-like hormone signaling and thereby limits lifespan. However, the fact that *srab-21* knockdown does not prolong lifespan suggests that simply altering ligand activation of DAF-2 is an unlikely mechanism.

The identification of the ATP-dependent DNA helicase gene *him-*6 as a *die* gene that promotes hypoxic death is consistent with previous results. In our whole genome RNAi screen, we identified a large number of DNA processing genes whose knockdown phenotype is hypoxia resistance [19]. Indeed, nucleic acid binding and processing enzyme genes represented the largest functional category with several helicases among them. *him-6* is expressed primarily in the germline as assessed by microarray experiments comparing animals with and without a germline [24]. Thus, some expression in somatic cells is possible. Germ cells from *him-6* mutant animals have increased sensitivity to ionizing radiation, but surprisingly have decreased germ cell apoptosis after genotoxic stress [25]. Thus, it is possible that the hypoxia resistance is secondary to defects in apoptosis but this remains to be determined.

We and others have identified a significant number of genes that regulate hypoxic sensitivity [1], [19], [26], [27], [28], [29]. In no case, is there a complete understanding of the mechanism(s) whereby a gene protects the organism and its cells from hypoxic injury, and in most cases, as for the genes identified here, there is no mechanistic information at all. Future studies must necessarily focus on the most promising hypoxic sensitivity genes. Such genes would have clear mammalian orthologs, preferably with a considerable understanding of their cellular function already defined. Additionally, the most promising genes should be able to be modulated to control hypoxic injury without producing other side effects. Thus, studies such as the one described here are important in order to identify specific pathways to target for hypoxic protection.

## Materials and Methods

### Nematode Maintenance

For strain maintenance worms were cultured at 20° on nematode growth medium (NGM) agar plates seeded with OP50 bacteria [30]. The following strains were utilized in this study: N2 (wild-type), CB1370 *daf-2(e1370)* III, DR1565 *daf-2(m596)* III, DR1572 *daf-2(e1368)* III, and CF1038 *daf-16(mu86)* I. Strains were obtained from the *Caenorhabditis* Genetics Center funded by the NIH NCRR.

### Hypoxia Assay

Worms were subjected to hypoxia as described previously [1]. Briefly, each plate was washed into one 1.5 mL tube with 1 mL of M9 buffer (22 mM KH_2_PO_4_, 22 mM Na_2_HPO_4_, 85 mM NaCl, 1 mM MgSO_4_) [30]. Worms were allowed to settle by gravity, and 800 µL of M9 was removed. The tubes were then placed in the anaerobic chamber (Forma Scientific) at 26.5° for 20, 22, or 24 hours, depending on the assay. Following the hypoxic insult, worms were placed on NGM plates spotted with OP50 bacteria using glass Pasteur pipets and recovered at 20° for 16−20 hours prior to scoring. Animals were scored as dead if no pharyngeal pumping or spontaneous or evoked movement (touching with a platinum wire) was observed. A minimum of triplicate plates for each trial and three trials/RNAi were scored.

### Feeding RNAi

One-generation feeding RNAi was performed as described previously [19], [31]. Briefly, two gravid adult worms were left on RNAi plates with 2 day old bacterial lawns for 3 hours to obtain 30−50 eggs per plate. RNAi plates were composed of NGM agar supplemented with 50 µg/ml carbenicillin and 1 mM IPTG and seeded with the appropriate RNAi bacterial strain cultured in 2xYT with 50 µg/ml carbenicillin, 10 µg/ml tetracycline and 0.8 mM IPTG. Animals were grown at 20°C to young adulthood, one day past the L4 stage and then phenotyped.

### Lifespan Assays

Lifespan assays were performed either on OP50 bacteria (Figure 1C) or on RNAi bacteria containing the L4440 empty vector control plasmid or the particular RNAi plasmid (Figure 5). Gravid adult worms were allowed to lay eggs for three hours onto OP50 or RNAi bacteria and picked off. The animals were cultured at 20°C until reaching the L4 stage and were then placed at a density of 30 worms/plate. For the *daf-2* allele lifespan data, the L4 animals were transferred to fresh NGM plates seeded with OP50. For the *die* gene RNAi lifespan experiments, L4 animals were transferred to new RNAi plates. Worms were transferred daily while egg-laying and transferred every three days thereafter onto plates with freshly seeded OP50 or RNAi bacteria. Worms were scored daily for survival and censored for bagging, exploding, and crawling off the plate.

### Thermotolerance Assay

Thermotolerance assays were performed in a 37° water bath. Young adult worms, one day post L4 stage, were washed into tubes using M9 buffer and allowed to settle by gravity. Excess M9 was removed to 100 µL. M9 buffer preheated to 37° was added to each tube to a final volume of 500 µL. The tubes were incubated in the water bath according to the time course. Worms were removed from the tubes at the appropriate time using a glass Pasteur pipette and allowed to recover at 20°C for 16−20 hours on NGM plates spotted with OP50 bacteria and then scored for survival.

### Dauer Arrest Assay

Ten gravid adult animals were allowed to lay eggs for one hour and picked off. Plates containing 30−50 eggs were put at 20°C, 23°C or 25°C. Animals were scored for dauer arrest when the non-dauer animals reached adulthood, 72 h or 96 h later. The fraction of non-adult animals that were partial dauers or arrested non-dauer larvae was not formally scored; however, the large majority of the non-adult animals were true dauers for all three strains as previously reported [13].

### cDNA Microarrays

Animals from a synchronous egg lay were cultured at 20°C on NGM plates seeded with OP50, and total RNA was isolated from L4 staged *daf-2(e1370)*, *daf-2(m596)* and *daf-2(e1368)* animals using Trizol reagent (Invitrogen). Dauer animals were separated from L4 larvae by differential pelleting in M9. RNA quality was measured using Nanodrop-1000 spectrophotometer (ThermoScientific). High quality RNA samples (OD 260/280>1.9) were used as inputs for microarrays. Eight micrograms of RNA was subjected to reverse transcription and subsequent hybridization using 3DNA Array 350 Kit (Genisphere, Inc, Hatfield, PA). Cy3- and Cy5-labeled samples were hybridized to *C. elegans* whole genome oligonucleotide microarrays (Genome Sequencing Center, Washington University in St. Louis). Slides were scanned on a ScanArray 3000 (Perkin Elmer, Waltham, MA) at 10 micrometer resolution. Three biological replicates (independent RNA isolations), each with a technical replicate (dye swap) were performed for each condition. Raw microarray fluorescent intensity data were processed and analyzed with GeneSpring GX 7.3.1 software (Agilent Technologies, Santa Clara, CA). Each time point was subjected to Lowess normalization (per spot and per array) separately. A flag filter was applied to include all genes that hybridized in three or more trials which narrowed the gene list to 14,317. One-way ANOVA (p<0.01) was performed to find genes that were statistically different between *e1370* and the other two *daf-2* alleles, *m596* and *e1368*. This gene list included 182 genes. Raw data have been deposited in the NCBI Gene Expression Omnibus database (http://www.ncbi.nlm.nih.gov/geo/), accession number GSE18601.

## Supporting Information

Table S1 List of genes upregulated in *daf-2*(e1370).
(0.06 MB XLS)

Table S2 Lifespan analysis of *die* gene RNAi knockdown.
(0.03 MB XLS)

Figure S1 Comparison of normoxic and hypoxic lethality. Young adult N2 animals were incubated at 28 degrees in M9 buffer in either an incubator with room air atmosphere (normoxia) or in an incubator with <0.3% oxygen (hypoxia). The % of dead animals was scored after recovery from various incubation times. Each point represents the mean +/− sd of a minimum of two trials with at least 16 animals/trial.
(2.48 MB TIF)
