# Supplementary figures and images for: Divergent Mechanisms Controlling Hypoxic Sensitivity and Lifespan by the DAF-2/Insulin/IGF-Receptor Pathway
Source: PLoS One. 2009 Nov 20;4(11):e7937. doi: 10.1371/journal.pone.0007937 (PMC2775958; doi:10.1371/journal.pone.0007937)

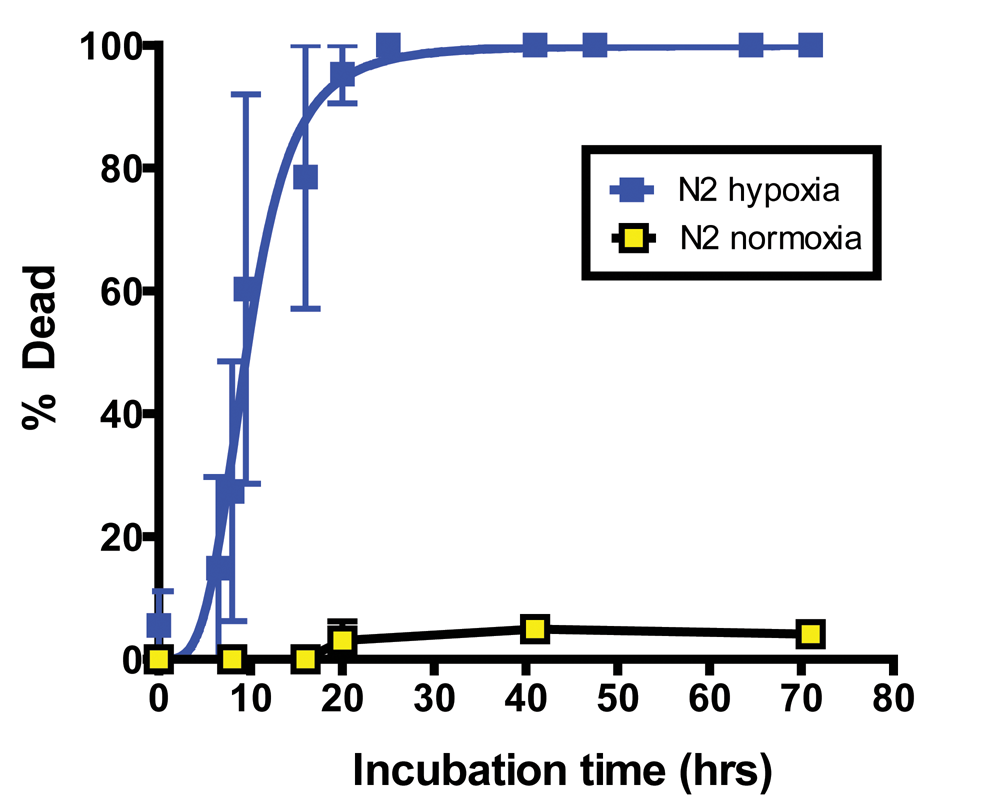

Supplement: Figure S1 — Comparison of normoxic and hypoxic lethality. Young adult N2 animals were incubated at 28 degrees in M9 buffer in either an incubator with room air atmosphere (normoxia) or in an incubator with <0.3% oxygen (hypoxia). The % of dead animals was scored after recovery from various incubation times. Each point represents the mean +/− sd of a minimum of two trials with at least 16 animals/trial. (2.48 MB TIF) [file pone.0007937.s003.tif]
